# Profile of the multicenter cohort of the German Cancer Consortium’s Clinical Communication Platform

**DOI:** 10.1007/s10654-023-00990-w

**Published:** 2023-04-05

**Authors:** Daniel Maier, Jörg Janne Vehreschild, Barbara Uhl, Sandra Meyer, Karin Berger-Thürmel, Melanie Boerries, Rickmer Braren, Viktor Grünwald, Boris Hadaschik, Stefan Palm, Susanne Singer, Martin Stuschke, David Juárez, Pierre Delpy, Mohamed Lambarki, Michael Hummel, Cäcilia Engels, Stefanie Andreas, Nicola Gökbuget, Kristina Ihrig, Susen Burock, Dietmar Keune, Angelika Eggert, Ulrich Keilholz, Hagen Schulz, Daniel Büttner, Steffen Löck, Mechthild Krause, Mirko Esins, Frank Ressing, Martin Schuler, Christian Brandts, Daniel P. Brucker, Gabriele Husmann, Thomas Oellerich, Patrick Metzger, Frederik Voigt, Anna L. Illert, Matthias Theobald, Thomas Kindler, Ursula Sudhof, Achim Reckmann, Felix Schwinghammer, Daniel Nasseh, Wilko Weichert, Michael von Bergwelt-Baildon, Michael Bitzer, Nisar Malek, Öznur Öner, Klaus Schulze-Osthoff, Stefan Bartels, Jörg Haier, Raimund Ammann, Anja Franziska Schmidt, Bernd Guenther, Melanie Janning, Bernd Kasper, Sonja Loges, Stephan Stilgenbauer, Peter Kuhn, Eugen Tausch, Silvana Runow, Alexander Kerscher, Michael Neumann, Martin Breu, Martin Lablans, Hubert Serve

**Affiliations:** 1 University Hospital Frankfurt, Frankfurt, Germany; 2 German Cancer Consortium (DKTK), Partner Site Frankfurt and German Cancer Research Center (DKFZ), Heidelberg, Germany; 3 Department of Internal Medicine I, University Hospital of Cologne, Cologne, Germany; 4 German Centre for Infection Research (DZIF), Partner Site Bonn-Cologne, Cologne, Germany; 5 University Hospital Munich, LMU Munich, Munich, Germany; 6 German Cancer Consortium (DKTK), Partner Site Munich and German Cancer Research Center (DKFZ), Heidelberg, Germany; 7 Faculty of Medicine, Institute of Medical Bioinformatics and Systems Medicine, Medical Center, University of Freiburg, Freiburg, Germany; 8 German Cancer Consortium (DKTK), Partner Site Freiburg and German Cancer Research Center (DKFZ), Heidelberg, Germany; 9 School of Medicine, Technical University Munich, Munich, Germany; 10 West German Cancer Center, University Hospital Essen, Essen, Germany; 11 German Cancer Consortium (DKTK), Partner Site Essen and German Cancer Research Center (DKFZ), Heidelberg, Germany; 12 University Medical Center of the Johannes Gutenberg University, Mainz, Germany; 13 German Cancer Consortium (DKTK), Partner Site Mainz and German Cancer Research Center (DKFZ), Heidelberg, Germany; 14 German Cancer Research Center (DKFZ), Federated Information Systems, Heidelberg, Germany; 15 German Cancer Consortium (DKTK), Partner Site Heidelberg and German Cancer Research Center (DKFZ), Heidelberg, Germany; 16 Charité Universitätsmedizin Berlin, Berlin, Germany; 17 German Cancer Consortium (DKTK), Partner Site Berlin and German Cancer Research Center (DKFZ), Heidelberg, Germany; 18 University Hospital and Faculty of Medicine Carl Gustav Carus, Technische Universität Dresden, Dresden, Germany; 19 German Cancer Consortium (DKTK), Partner Site Dresden and German Cancer Research Center (DKFZ), Heidelberg, Germany; 20 Department of Medicine I, Faculty of Medicine, Medical Center, University of Freiburg, Freiburg, Germany; 21 Center for Personalized Medicine, Eberhard-Karls University of Tübingen, Tübingen, Germany; 22 German Cancer Consortium (DKTK), Partner Site Tübingen and German Cancer Research Center (DKFZ), Heidelberg, Germany; 23 University Medical Center Hamburg-Eppendorf, Hamburg, Germany; 24 Comprehensive Cancer Center Hannover (Claudia von Schilling-Zentrum), Hannover Medical School, Hannover, Germany; 25 DKFZ-Hector Cancer Institute at the University Medical Center Mannheim, Mannheim, Germany; 26 Mannheim University Medical Center, University of Heidelberg, Mannheim, Germany; 27 Department of Personalized Medical Oncology (A420), DKFZ German Cancer Research Center, Heidelberg, Germany; 28 Comprehensive Cancer Center Ulm, Ulm, Germany; 29 Neu-Ulm University of Applied Sciences, Neu-Ulm, Germany; 30 University Hospital of Würzburg, Würzburg, Germany; 31 Frankfurt Cancer Institute, Frankfurt, Germany

**Keywords:** Cohort profile, Pan-cancer, Real-world data, Federated analysis, German Cancer Consortium

## Abstract

Treatment concepts in oncology are becoming increasingly personalized and diverse. Successively, changes in standards of care mandate continuous monitoring of patient pathways and clinical outcomes based on large, representative real-world data. The German Cancer Consortium’s (DKTK) Clinical Communication Platform (CCP) provides such opportunity. Connecting fourteen university hospital-based cancer centers, the CCP relies on a federated IT-infrastructure sourcing data from facility-based cancer registry units and biobanks. Federated analyses resulted in a cohort of 600,915 patients, out of which 232,991 were incident since 2013 and for which a comprehensive documentation is available. Next to demographic data (i.e., age at diagnosis: 2.0% 0–20 years, 8.3% 21–40 years, 30.9% 41–60 years, 50.1% 61–80 years, 8.8% 81+ years; and gender: 45.2% female, 54.7% male, 0.1% other) and diagnoses (five most frequent tumor origins: 22,523 prostate, 18,409 breast, 15,575 lung, 13,964 skin/malignant melanoma, 9005 brain), the cohort dataset contains information about therapeutic interventions and response assessments and is connected to 287,883 liquid and tissue biosamples. Focusing on diagnoses and therapy-sequences, showcase analyses of diagnosis-specific sub-cohorts (pancreas, larynx, kidney, thyroid gland) demonstrate the analytical opportunities offered by the cohort’s data. Due to its data granularity and size, the cohort is a potential catalyst for translational cancer research. It provides rapid access to comprehensive patient groups and may improve the understanding of the clinical course of various (even rare) malignancies. Therefore, the cohort may serve as a decisions-making tool for clinical trial design and contributes to the evaluation of scientific findings under real-world conditions.

## Introduction

Established in 2012, the German Cancer Consortium (DKTK) is an alliance connecting university medical center-based comprehensive cancer centers (CCC) and the German Cancer Research Center (DKFZ) with the goal to foster translational cancer research [1]. For that purpose, the Clinical Communication Platform (CCP), a key instrument for cross-center networked research, operates a federated data warehouse system populated with real-world data (RWD) of patients and biosamples. With increasing data volume, the CCP’s functionality shifts from finding and recruiting patients for clinical trials to compiling patient cohorts and using this data directly for research purposes. Activities in clinical data science and the rising potential of machine learning algorithms promise enhanced (translational) value of such RWD [2–6]. To invigorate respective research activities in clinical epidemiology and outcomes research in Germany and to offer a joint interface for international collaboration, we introduce the pan-cancer multicenter clinical cohort of the DKTK’s CCP.

With initially nine sites, the CCP grew beyond its original borders of the DKTK-network, and currently connects fourteen university hospital-based cancer centers, including the largest CCCs designated by the German Cancer Aid (DKH).^1^ The cohort is considered representative for a specific section of real-world cancer care in Germany as it mirrors the most advanced care standards of tertiary care centers with pioneering potential for other hospitals.

In this cohort profile, we describe the technical basis of the CCP-infrastructure as well as methodological aspects of the here-applied federated analysis. Our analysis details information about patient demographics, cohort growth and disease-specific statistics. For a better understanding of the available data quality and quantity, this cohort overview is complemented with an in-depth inquiry of four disease-specific sub-cohorts (pancreatic cancer, laryngeal cancer, kidney cancer and cancer of the thyroid gland), for which we provide exemplary diagnosis- and treatment-related analyses as *in-silico* validation instrument. Finally, we discuss how future projects can use the cohort data to tap their translational potential.

## Methods

### Ethics and patient consent

The cohort profile is the result of a federated analysis of patient data that required only aggregated, non-personal information to be exchanged among the sites and allowed all personal data to remain safely within each hospital. All patients were treated and observed according to institutional guidelines. In this setting, no ethics vote or informed consent is legally required. Additionally, ten participating centers approved the project (ethics committees of seven centers independently approved the project, three more centers accepted the initial vote).

### Data infrastructure: federated concept of the CCP

**Fig. 1:**
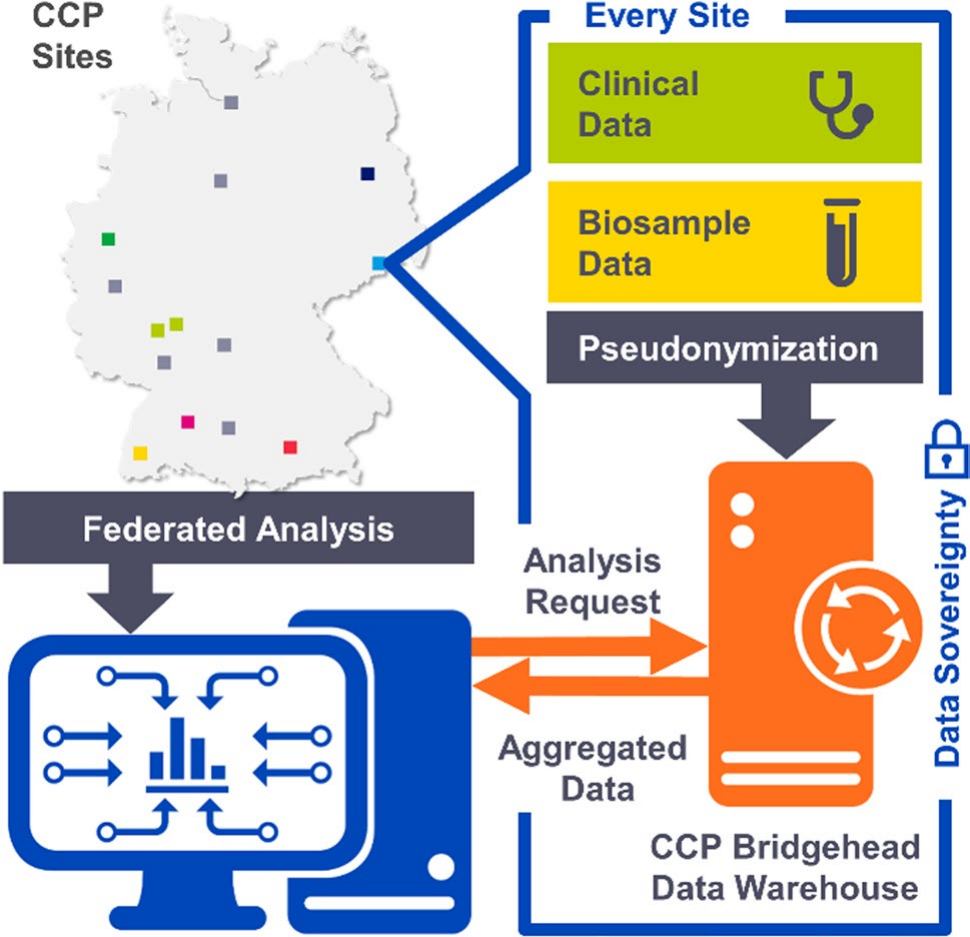
CCP-Bridgehead infrastructure and federated analysis

The cohort is based on the CCP’s federated system of so-called *bridgeheads*, which have been customized for the collection, storage and analysis of multi-center RWD [8]. Bridgeheads serve as local data-hubs which facilitate effective cooperation and the exchange of (pseudonymized) data. For the participating university hospitals, this infrastructure guarantees sovereignty over their data [9]. Local IT administration safeguards data entering and leaving their servers and ensures that local rules and regulations are properly applied. Figure 1 illustrates the federated concept of the CCP’s data infrastructure. The institutions who contributed data to the cohort profile are Charité Universitätsmedizin Berlin, Hospital of the Carl Gustav Carus Technical University Dresden, University Hospital Essen, University Hospital Frankfurt, University Hospital Freiburg, University Medical Center of the Johannes Gutenberg University Mainz, Hospital of the Technical University Munich, Hospital of the Ludwig Maximilians University Munich, Hospital of the Eberhard-Karls University Tübingen, University Medical Center Hamburg-Eppendorf, Comprehensive Cancer Center Hannover, Mannheim University Medical Center, Comprehensive Cancer Center Ulm, and University Hospital Würzburg.

Bridgeheads hold a specified set of data in a standardized and extensible format covering the most significant information of the patients’ diagnoses and events over their course of disease and treatment. In addition to this clinical information, the availability of liquid or tissue biosamples is also covered. The data set builds on the Unified Basic Oncological Data Set (German: *Einheitlicher Onkologischer Basisdatensatz*) defined and maintained by the Association of German Tumor Centers (German: *Arbeitsgemeinschaft Deutscher Tumorzentren*, short ADT), the Association of Population Based Cancer Registries in Germany (German: *Gesellschaft der epidemiologischen Krebsregister in Deutschland e.V.*) and the *Platform §65c* (a panel of experts consisting of one representative from each of the state cancer registries in Germany). For reportable events, healthcare providers are required by law to report ADT-formatted data to the clinical cancer registries of the German federal states. While many other routine documentation data sources provide only diagnoses, treatments or outcomes, the CCP’s clinical data provides patient and diagnosis-related as well as treatment and outcome-related information. For example, the CCP data allows to map the treatment modalities of patients with primary cancer of the colon who developed liver metastases after first-line systemic therapy and to successively analyze their overall survival.

Bridgeheads support various methods of local or cross-site pseudonymization with fault-tolerant, privacy-preserving record linkage [10, 11]. This allows to extend the bridgeheads with data from other sources, e.g., studies conducted within the DKTK or the primary routine documentation systems within each hospital. The federated infrastructure of the CCP comes with the advantage to potentially join further data elements from already developed sources (e.g., dose information for substances administered in systemic therapy from the source tumor registry data) or to connect adjacent data sources containing, e.g., laboratory parameters or radiological imaging.

### Data quality assessment

RWD research is often accompanied by data quality issues [12–14], for example, when patient data are missing or not documented at all. This reduces the power of data analyses and may lead to biased results if the missingness is not at random, for instance, when histopathological information is more often missing in patients because of guidelines or for technical reasons. Concerning the cohort presented here, it is important to note, that patient data were derived from DKTK-sites and other university hospital-based cancer centers, representing a specific selection of tertiary cancer care in Germany.

When working with data from 14 different sites, some degree of heterogeneity regarding collection and annotation of data can be expected. To monitor and, if necessary, improve data quality, especially with respect to completeness and syntactic validity, the CCP bridgeheads are connected to a central metadata repository (MDR) that contains the definitions of the agreed upon data elements. The MDR is used to automatically generate standardized quality reports that are used for cross-site comparative data quality assessments and to unveil data inconsistencies [15] such as invalid values for post-operative residual tumor status or the missingness of the mandatory ICD-coded diagnosis.

The here-presented data is facility-based data. As compared to German cancer registry data, the CCP data has similar but less complex demands for harmonization. Most importantly, within CCP harmonization processes only comprise a within-facility “best-of” information selection as compared to a more comprehensive “best-of” from concurrent sources as in registries. However, to keep comparability with registry data high, data management and processing is based on the standards set by common practice of the German cancer registries [16].

Additionally, we deemed as an essential requirement to have some information about the conditions under which documentation was conducted. We launched a survey among the cancer registry units of the participating sites; 13 out of 14 sites answered the email-administered questionnaire. Additionally, telephone calls were made to confer explanations if required. Most importantly, we asked whether the registry units established processes to check the validity, completeness, and the plausibility [16] of their data. While a detailed description of the survey’s findings is beyond the scope of this paper, it is important to state that the majority of the study sites (12 out of 13) conduct internal and software-based data plausibility and validity checks. Additionally, the respondent units indicated that 87.5% of cases are locally registered within the first five months after the event.

### Statistical analysis

For statistical analyses a federated procedure was applied: Instead of transferring patient data from multiple sites to a central database to conduct statistical analysis, analyses were performed at local facilities. Only aggregated and non-disclosive data were transferred for manual cross-site result aggregation. This approach is conceptually based on what is proposed by federated learning (FL) software implementations following the principle ‘not bringing the data to the analysis, but bringing analysis to the data’ [17, 18]. Following a coordinated statistical analysis plan, an analysis script was designed in the statistical programming language R (Version 4.0.4) [19]. At each site, the script was executed on a local copy of the CCP-bridgehead data. Data processing and successive analyses were conducted between December 2021 and April 2022.

The statistical analysis focused on overview figures characterizing the patient cohort stratified by disease (according to the tenth version of International Statistical Classification of Diseases and Related Health Problems, ICD-10), i.e., counts of respective primary diagnoses and available biosamples, mean and standard deviation of age at primary diagnosis, the percentage of patients who survived a five-year period after diagnosis (5-year overall-survival) including 95%-confidence intervals, the percentage of female patients as an indicator for gender distribution and an estimator for cohort coverage. Coverage estimation was conducted to evaluate how many patients with respective diagnosis received examination or treatment in medical centers of the participating sites. Coverage estimation is calculated by dividing the sum of cohort patients with the respective diagnosis by the incident cases registered in the cancer registry database of the German Center for Cancer Registry Data (Zentrum für Krebsregisterdaten, https://www.krebsdaten.de).^2^ The coverage measure estimates whether the cohort can be regarded representative for the population of cancer patients in Germany. High coverage (values beyond the 90th quantile of the coverage distribution, i.e., 13.3%) is interpreted as an indicator for the diagnosis-specific patient group to be overrepresented in the cohort as compared to the general population of cancer patients in Germany.

As an *in-silico* validation,^3^ and in order to illustrate the depth of the cohort data, four disease-specific sub-cohorts were formed (pancreatic cancer, laryngeal cancer, kidney cancer and cancer of the thyroid gland), for which an in-depth descriptive and visual analysis was conducted. The sub-cohorts depict frequent diseases from different organ systems (digestive, respiratory, genitourinary and endocrine) with different therapeutic approaches and outcomes. The frequency of patients with specified localization and the stage at diagnosis combination is depicted in so-called Voronoi treemaps [21]. Additionally, the sequences-of-therapies stratified for stage at diagnosis are visualized as alluvial diagrams.

## Results

### Cohort overview

**Fig. 2:**
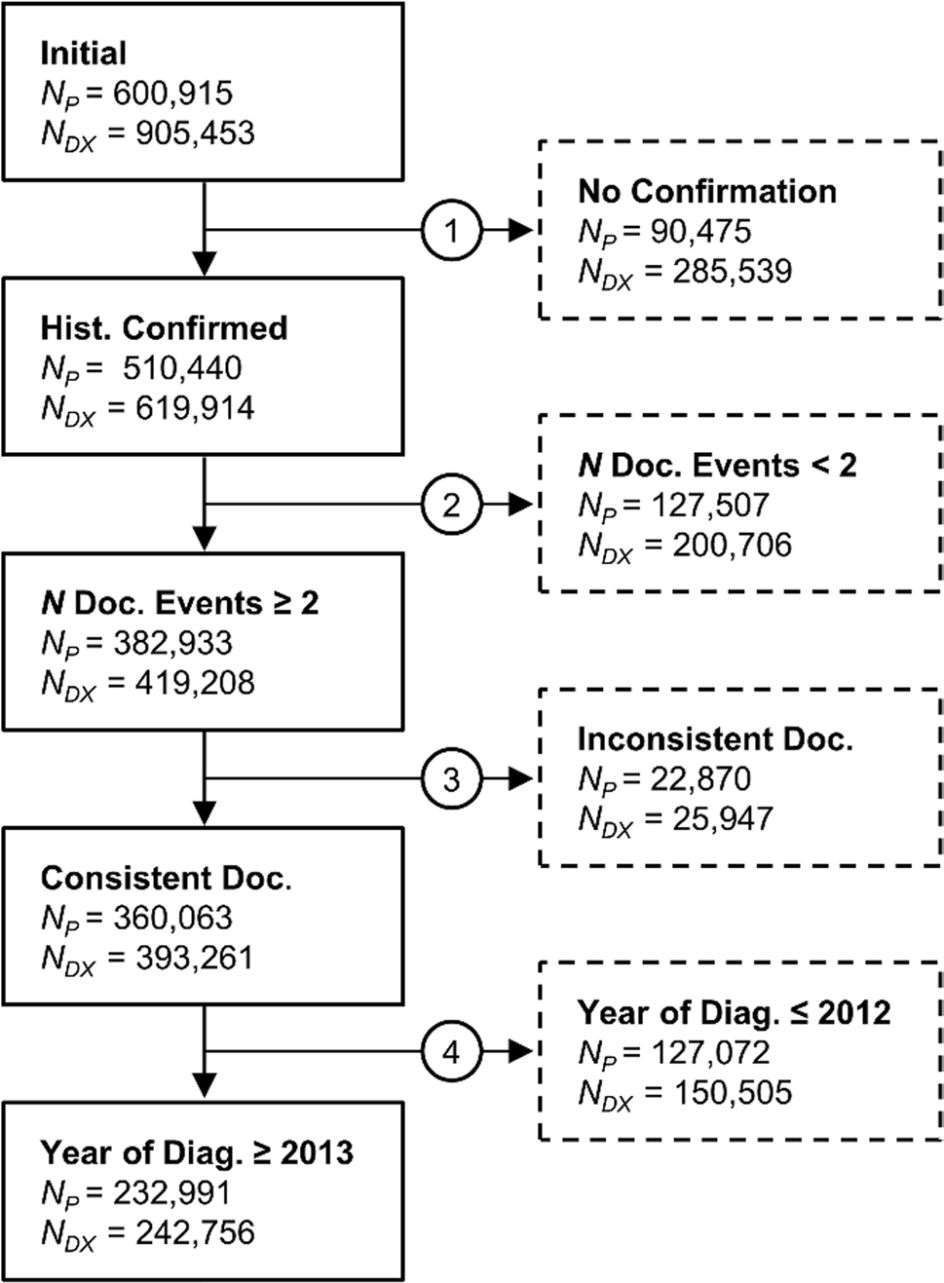
Assembly of the cohort through a four-step process to ensure data quality. The numbers of patients (*N*_*P*_) and diagnoses (*N*_*DX*_) at each filter step that are included (solid lined boxes) and excluded (dashed lined boxes) are indicated

Local bridgeheads from fourteen participating cancer centers in Germany comprise data of *N*_*P*_ = 600,915 patients and *N*_*DX*_ = 905,453 diagnoses. As illustrated in Fig. 2 a four-step data quality assuring process was applied to assemble the cohorts’ patients with (1) a histologically confirmed primary diagnosis (excluded: *N*_*P*_ = 90,475),^4^ (2) a minimum of two documented visits, defined as examination or anti-cancer-treatment events (excluded: *N*_*P*_ = 127,507 patients), (3) logically consistent event dates (e.g. primary diagnosis had to be no later than first treatment, excluded: *N*_*P*_ = 22,870)^5^ and (4) year of diagnosis was 2013 or later (excluded: *N*_*P*_ = 127,072). The latter starting date was chosen because in 2013 the Cancer Screening and Registry Act (German: *Krebsfrüherkennungs- und Registergesetz*, KFRG) entered into force which led to the establishment of a comprehensive cancer documentation practice.^6^ These exclusion criteria led to a final cohort size of *N*_*P*_ = 232,991 (*N*_*DX*_ = 242,756) or 39% of the total cohort.

**Fig. 3:**
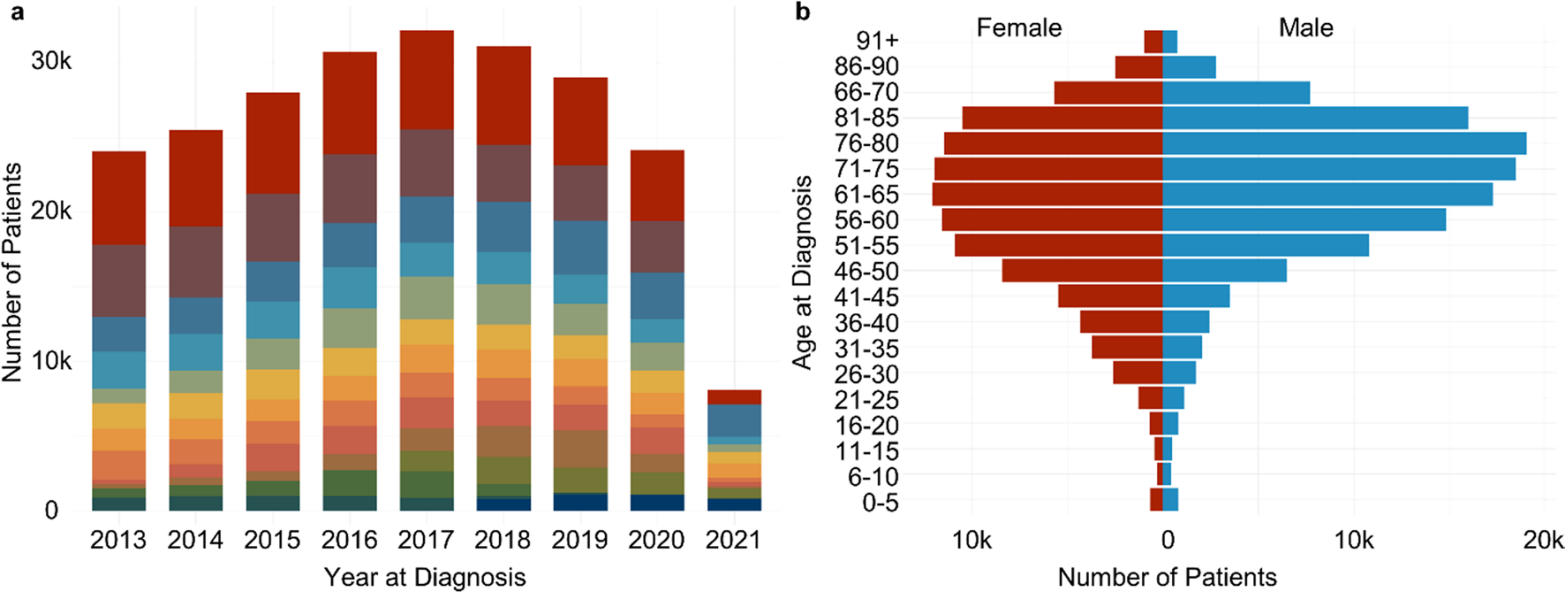
Distribution of patient numbers in the cohort **a** over time (year of diagnosis 2013–2021) and by participating medical center (color-coded) and **b** regarding demographic factors (age at diagnosis, gender status)

Figure 3a illustrates the distribution of the count of patients by all fourteen participating medical centers. The median of contributed patients is *N*_*P*_ = 13,194.5 (interquartile range (IQR) = [7802; 18,285]), varying markedly between sites, ranging from *N*_*P*_ = 51,326 at the largest center to *N*_*P*_ = 3632 patients at the smallest center. Focusing on the year of diagnosis, an increasing patient count can be observed from *N*_*P*_ = 24,090 in 2013 to *N*_*P*_ = 32,191 in 2017 followed by a slight decrease until 2020 (*N*_*P*_ = 24,162) and a sharp decline thereafter (*N*_*P*_ = 8078 patients in 2021).

The cohort’s distribution of demographics, age at diagnosis and gender, are depicted in Fig. 3b. Overall, the cohort comprises more male (*N*_*P*_ = 127,543) than female patients (*N*_*P*_ = 105,425). Sixteen patients were coded with unknown (*N*_*P*_ = 12), other (*N*_*P*_ = 3) or missing (*N*_*P*_ = 1) gender status. Importantly, the age distribution differs between female and male patients. Due to the high prevalence of breast cancer in younger females (mean age at diagnosis = 59.3 (*SD* = 13.8)), the frequency distribution of female patients shows an earlier, steeper onset, than the frequency distribution for male patients (mean age at diagnosis = 68.0 (*SD* = 8.2)). In contrast, the most common cancer diagnosis in male patients is prostate cancer; a disease that more often affects older men. Also, more men than women are affected by highly prevalent cancer diagnoses such as lung cancer and colorectal cancer.

Table 1 provides an overview over the total number of patients with their primary diagnosis, the mean and standard deviation of age at diagnosis, the number of available biosamples, the percentage of female patients with the respective diagnosis and the estimated coverage with respect to the total number of incident cases in Germany.

**Table 1:** Cohort characteristics grouped by primary diagnosis

Primary diagnosis^a^	ICD-10 code	Number of patients (*N*)	Age at diagnosis (*Mean* (*SD*))	Biosamples (*N*)	Female patients (%)	Patients (%) with 5-year OS (95% CI)^b^	Est. cover-age^c^
C00-C14: Lip, oral cavity and pharynx							
Tonsil	C09	1922	63.2 (10.0)	1267	26.4	71.1 (68.1–74.2)	11.3
Tongue, other and ill-defined parts	C02	1849	62.7 (13.5)	916	38.7	64.8 (61.4–68.3)	10.8
Oropharynx	C10	1517	63.4 (9.1)	933	25.2	55.5 (52.0–59.3)	10.1
Floor of mouth	C04	1467	62.2 (10.0)	757	27.9	59.5 (55.5–63.6)	11.8
Hypopharynx	C13	1192	64.4 (9.4)	651	14.7	37.9 (34.0–42.3)	9.7
Base of tongue	C01	1090	63.4 (9.9)	733	23.4	62.9 (58.6–67.5)	10.2
Gum	C03	1053	68.1 (12.6)	529	44.1	61.8 (57.3–66.6)	14.3
Parotid glands	C07	640	63.7 (17.9)	126	40.9	60.4 (54.7–66.7)	8.2
Mouth, other and unspecified parts	C06	621	65.5 (13.3)	313	45.4	62.7 (57.4–68.4)	9.6
Palate	C05	598	62.2 (12.3)	223	35.6	64.5 (59.0–70.6)	10.0
Nasopharynx	C11	489	53.4 (15.8)	101	28.0	70.3 (65.0–76.1)	13.1
Lip	C00	261	72.9 (13.3)	40	33.7	64.7 (55.1–76.0)	4.3
Pyriform sinus	C12	206	66.0 (9.4)	215	18.4	48.2 (39.5–58.9)	7.9
Salivary glands	C08	191	60.8 (16.1)	100	41.9	68.1 (59.5–77.8)	9.2
Mouth and pharynx	C14	115	64.8 (10.2)	64	23.5	40.6 (28.3–58.1)	4.9
C15-C26: Digestive organs							
Colon	C18	6218	64.0 (14.3)	9254	42.9	57.9 (56.1–59.8)	1.7
Pancreas	C25	6009	64.9 (11.7)	7041	45.6	31.5 (29.6–33.6)	3.6
Liver and bile duct	C22	5907	65.2 (12.3)	6750	26.4	36.9 (34.9–39.1)	7.0
Rectum	C20	4982	62.9 (12.5)	8480	34.0	61.9 (59.8–64.1)	2.9
Stomach	C16	4221	62.8 (12.9)	7044	33.1	46.3 (43.9–48.7)	3.0
Esophagus	C15	2940	65.1 (10.7)	1160	22.0	37.6 (34.7–40.7)	4.5
Small intestine	C17	1385	61.5 (12.6)	1197	46.3	81.5 (78.7–84.4)	6.0
Biliary tract	C24	1241	67.3 (11.1)	1020	36.6	31.4 (27.5–35.9)	3.7
Anal canal	C21	885	62.4 (11.9)	250	62.0	69.6 (65.2–74.3)	4.5
Gallbladder	C23	320	66.0 (11.2)	215	62.5	27.0 (20.1–36.3)	2.2
Rectosigmoid junction	C19	143	63.0 (14.0)	121	33.6	66.3 (57.1–76.9)	1.2
Digestive organs, other and ill-defined location	C26	44	61.7 (13.4)	26	38.6	44.1 (27.6–70.5)	0.5
C30-C39: Respiratory and intrathoracic organs							
Lung	C34	15,575	65.8 (10.2)	6745	40.7	37.0 (35.8–38.2)	3.0
Larynx	C32	2931	65.6 (10.5)	869	15.0	65.0 (62.4–67.6)	9.1
Nasal cavity and middle ear	C30	664	62.0 (13.9)	257	41.1	72.1 (66.9–77.8)	11.0
Accessory sinuses	C31	429	61.2 (14.2)	157	31.9	61.0 (54.2–68.6)	11.8
Thymus	C37	236	60.0 (13.3)	93	44.1	61.8 (51.5–74.2)	10.4
Heart, mediastinum and pleura	C38	132	50.4 (20.5)	69	27.3	40.4 (29.7–54.8)	4.5
Trachea	C33	24	60.4 (15.5)	23	50.0	63.0 (42.9–92.6)	3.4
Respiratory and intrathoracic system, other and ill-defined sites	C39	< 5	–	–	–	–	1.2
C40-C41: Bone and articular cartilage							
Bone and articular cartilage, other and unspecified sites	C41	664	45.8 (22.7)	899	42.3	71.4 (66.0–77.1)	14.5
Bone and articular cartilage of limbs	C40	633	40.6 (22.9)	737	43.1	77.3 (72.4–82.5)	19.7
C43: Malignant melanoma							
Malignant melanoma	C43	13,964	63.0 (15.8)	3657	44.0	80.3 (79.1–81.4)	6.9
C45-C49: Mesothelial and soft tissue							
Connective and soft tissue	C49	3907	57.0 (19.8)	3852	43.2	68.5 (66.1–71.0)	13.4
(Retro-)peritoneum	C48	553	59.2 (14.7)	763	55.7	54.8 (48.4–62.2)	6.8
Mesothelioma	C45	340	68.0 (13.1)	903	23.8	28.0 (21.2–37.2)	2.3
Peripheral nerves	C47	203	34.0 (26.8)	99	40.9	60.0 (49.5–72.7)	13.1
Kaposi sarcoma	C46	150	60.5 (17.3)	49	18.0	85.4 (77.0–94.6)	10.8
C50-C58: Breast and female genital organs							
Breast	C50	18,409	59.3 (13.8)	18,513	98.9	84.6 (83.8–85.4)	2.9
Ovary	C56	3548	57.9 (14.9)	1819	100.0	60.3 (57.8–62.9)	5.2
Corpus uteri	C54	2900	64.2 (12.0)	785	100.0	65.4 (62.5–68.3)	3.1
Cervix uteri	C53	2592	49.9 (14.1)	340	100.0	69.0 (66.3–71.8)	6.4
Vulva	C51	1168	65.8 (14.2)	104	100.0	64.6 (60.5–68.9)	3.9
Female genital organs, other and unspecified sites	C57	341	62.3 (12.3)	156	100.0	66.0 (58.2–74.8)	4.3
Vagina	C52	234	64.1 (14.3)	88	100.0	45.2 (36.3–56.3)	5.4
Uterus, unspecified part	C55	199	58.8 (13.8)	99	100.0	57.8 (48.7–68.6)	5.1
Placenta	C58	28	36.8 (9.4)	2	100.0	21.4 (10.5–43.6)	14.8
C60-C63: Male genital organs							
Prostate	C61	22,523	68.0 (8.2)	7381	0.0	82.7 (81.8–83.6)	4.1
Testis	C62	1652	36.4 (12.1)	462	0.1	92.1 (89.6–94.6)	4.3
Penis	C60	329	66.7 (11.9)	145	0.0	62.2 (54.4–71.0)	3.9
Male genital organs, other and unspecified sites	C63	72	58.7 (18.5)	27	0.0	79.4 (66.1–95.4)	5.6
C64-C68: Urinary tract							
Bladder	C67	5279	69.2 (11.3)	2265	24.7	46.8 (44.7–49.0)	3.4
Kidney	C64	4271	61.7 (16.1)	2495	30.6	65.9 (63.8–68.0)	3.2
Renal pelvis	C65	427	70.6 (9.9)	108	37.9	44.2 (37.5–52.2)	3.5
Ureter	C66	254	70.5 (10.7)	35	28.7	41.8 (32.6–53.6)	3.8
Urinary organs, other and unspecified sites	C68	143	69.8 (12.2)	21	21.0	41.6 (29.4–58.7)	2.2
C69-C72: Eye, brain and CNS							
Brain	C71	9005	55.2 (19.1)	10,460	41.8	42.0 (40.5–43.7)	14.3
Eye	C69	2538	59.6 (19.7)	274	47.8	80.1 (77.1–83.1)	36.2
Spinal cord and other CNS	C72	341	46.2 (18.9)	156	48.7	87.5 (82.2–93.1)	16.8
Meninges	C70	63	61.4 (17.5)	43	50.8	70.9 (57.4–87.6)	4.5
C73-C75: Endocrine glands							
Thyroid gland	C73	4696	48.8 (16.6)	1195	66.9	92.7 (91.5–93.9)	7.8
Adrenal gland	C74	288	36.2 (26.8)	254	55.9	60.6 (51.8–71.0)	10.5
Endocrine glands	C75	188	47.4 (23.1)	36	40.4	73.4 (63.1–85.4)	15.7
C76-C80: Ill-defined, secondary and unspecified sites							
Carcinoma with unknown primary site	C80	2541	64.4 (13.4)	1151	41.6	35.6 (32.8–38.7)	2.7
Other and ill-defined sites	C76	248	61.1 (17.0)	47	35.9	54.2 (46.5–63.1)	3.4
C81-C96: Lymphoid and hematopoietic neoplasms							
Lymphoma, non-follicular	C83	5837	62.3 (17.0)	1851	39.2	64.7 (62.9–66.6)	7.0
Myeloid leukemia	C92	4396	56.9 (17.6)	8961	44.4	52.5 (50.4–54.6)	9.0
Multiple myeloma	C90	3657	63.5 (11.2)	1337	39.4	64.6 (62.2–67.0)	5.8
Lymphoid leukemia	C91	2299	41.2 (26.7)	1411	38.2	75.1 (72.5–77.7)	3.7
Hodgkin-lymphoma	C81	1949	38.6 (18.8)	513	40.6	91.3 (89.5–93.2)	8.7
Lymphoma, follicular	C82	1481	61.6 (13.2)	344	48.4	82.8 (80.1–85.5)	5.1
Mature T/NK-cell-lymphoma	C84	901	58.5 (17.5)	228	34.1	66.8 (62.5–71.3)	7.5
Non-Hodgkin lymphoma, unspecified	C85	717	56.4 (19.8)	205	47.8	76.6 (72.3–81.1)	2.7
Malignant immunoproliferative disease	C88	655	61.6 (15.6)	117	46.0	87.8 (84.1–91.7)	5.5
Monocytic leukemia	C93	385	59.8 (19.9)	669	41.0	44.7 (38.1–52.4)	5.4
T/NK-cell-lymphoma, other	C86	320	59.5 (16.4)	69	40.3	57.1 (49.4–66.2)	10.2
Lymphoid and hematopoietic tissue, other and unspecified neoplasms	C96	216	36.8 (26.4)	88	34.7	78.7 (71.2–87.1)	10.0
Leukemia, other of specified cell type	C94	108	57.9 (22.0)	29	38.0	35.1 (24.5–50.4)	8.5
Leukemia, other of unspecified cell type	C95	82	44.2 (24.1)	83	37.8	52.0 (40.7–66.3)	1.4

^a^According to ICD-10, including codes with digits after decimal point; C44, D00-D09, D10-D36 and D37-D38 diagnosis codes are not displayed

^b^Overall-survival (OS), including 95%-confidence intervals (CI)

^c^Ratio of total cohort patients to incident cases in Germany

The cohort covers diagnoses of solid cancers (*N*_*DX*_ = 172,190) from all organ systems (lip, oral cavity and pharynx: *N*_*DX*_ = 13,211; digestive organs: *N*_*DX*_ = 34,295; respiratory and intrathoracic Organs: *N*_*DX*_ = 19,993; bone and articular cartilage: *N*_*DX*_ = 1297; malignant melanoma: *N*_*DX*_ = 13,964; mesothelial and soft tissue: *N*_*DX*_ = 5153; breast and female genital organs: *N*_*DX*_ = 29,419; male genital organs: *N*_*DX*_ = 24,576; urinary tract: *N*_*DX*_ = 10,374; eye, brain and CNS: *N*_*DX*_ = 11,947; endocrine glands: *N*_*DX*_ = 5172; ill-defined and unspecified: *N*_*DX*_ = 2789) as well as a wide spectrum of malignancies of the hematopoietic and lymphatic system (*N*_*DX*_ = 23,003).

More specifically, the cohort’s ten most frequent diagnoses are prostate cancer (*N*_*DX*_ = 22,523, cohort rank 1 vs. population rank 2), breast cancer (*N*_*DX*_ = 18,409; cohort rank 2 vs. population rank 1), lung cancer (*N*_*DX*_ = 15,575; cohort rank 3 vs. population rank 3), malignant melanoma of the skin (*N*_*DX*_ = 13,964; cohort rank 4 vs. population rank 5), colon cancer (*N*_*DX*_ = 6218; cohort rank 6 vs. population rank 4), pancreatic cancer (*N*_*DX*_ = 6009; cohort rank 7 vs. population rank 7) and cancer of the bladder (*N*_*DX*_ = 5279; cohort rank 10 vs. population rank 8).^7^ Only malignant tumors of the brain (*N*_*DX*_ = 9005; cohort rank 5 vs. population rank 17), cancer of the liver and bile duct (*N*_*DX*_ = 5907; cohort rank 8 vs. population rank 13) and diffuse non-Hodgkin lymphoma (*N*_*DX*_ = 5837; cohort rank 9 vs. population rank 15) are among the ten most frequent diagnoses of the cohort deviating from the top ten cancer diagnoses in the population.

The median diagnosis-specific coverage is 5.7% (IQR = [3.7%; 10.1%]). The estimated coverage of many frequent diagnoses such as lung cancer (3.0%), breast cancer (2.9%) and prostate cancer (4.1%) lie within the IQR (cf. estimated coverage in Table 1). However, the coverage of neuro-oncological cancers such as malignant tumors of the brain (14.3%) and the eye (36.2%) as well as cancer of the spinal cord and other unspecified parts of the CNS (16.8%) lie beyond the 90th quantile of the coverage distribution (13.3%); but also rather rare entities such as malignant tumors of the bones and articular cartilage (14.5% and 19.7%) and cancer of endocrine glands (15.7%), the placenta (14.8%) or connective and soft tissue (13.4%) feature a high estimated coverage in the cohort.

### Analysis of diagnosis-specific sub-cohorts

In order to prove the validity of the data, we performed a disease- and therapy-related analysis that covers four sub-cohorts (pancreatic cancer, laryngeal cancer, kidney cancer and cancer of the thyroid gland), reproducing known cancer-specific traits of patients’ clinical courses. The cancer entities differ with respect to the distribution of stage at diagnosis and therapeutic approaches.

**Fig. 4:**
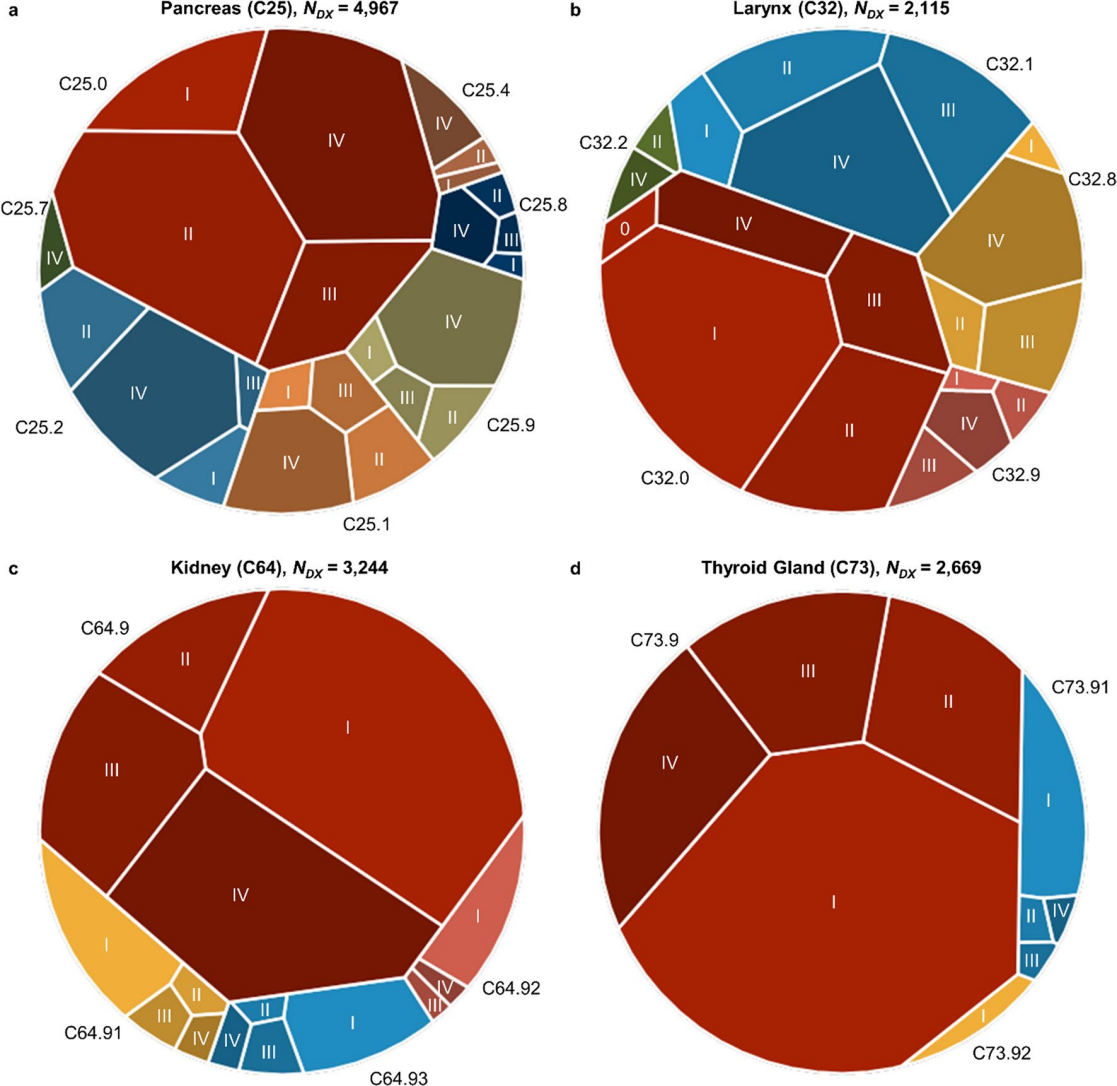
Frequency of UICC stage at time of diagnosis by specified tumor localization for cancer of the **a** pancreas, **b** larynx, **c** kidney and **d** thyroid gland. *Notes* number of diagnoses (*N*_*DX*_) according to ICD-10 (including codes with digits after decimal point); tumor localization-specific subtypes according to ICD-O (C25.0 head of pancreas, C25.1 body of pancreas; C25.2 tail of pancreas; C25.4 endocrine pancreas; C25.7 other parts; C25.8 overlapping lesion; C25.9 unspecified; C32.0 glottis; C32.1 supraglottis; C32.2 subglottis; C32.8 overlapping lesion; C32.9 unspecified; C64.9 kidney; C64.91 kidney, upper third; C64.92 kidney, middle third; C64.93 kidney, lower third; C73.9 thyroid gland unspecific; C73.91 lobe of thyroid gland; C73.92 isthmus of thyroid gland) are color-coded; UICC stage is coded by color intensity (darker color indicates the more advanced stage at the time of diagnosis); area size indicates relative frequency of a stage-localization combination

Voronoi treemaps (Fig. 4) illustrate the frequency of tumor location-specific subtype and its UICC (Union Internationale Contre le Cancer) stage at the time of diagnosis. Pancreatic cancer is most frequently detected in an advanced stage (UICC stage I: 11.3%, II: 31.0%, III: 11.8%, IV: 45.9%). For laryngeal cancer, the UICC stage at diagnosis is more homogenously distributed (UICC stage 0: 1.0%, I: 26.8%, II: 20.8%, III: 20.0%, IV: 31.4%). Cancers of the kidney and thyroid gland are often detected in an earlier stage (kidney cancer UICC stage at diagnosis: I: 51.2%, II: 7.6%, III: 15.4%, IV: 25.8%; cancer of the thyroid gland UICC stage at diagnosis I: 58.8%, II: 13.9%, III: 13.1%, IV: 14.3%).

**Fig. 5:**
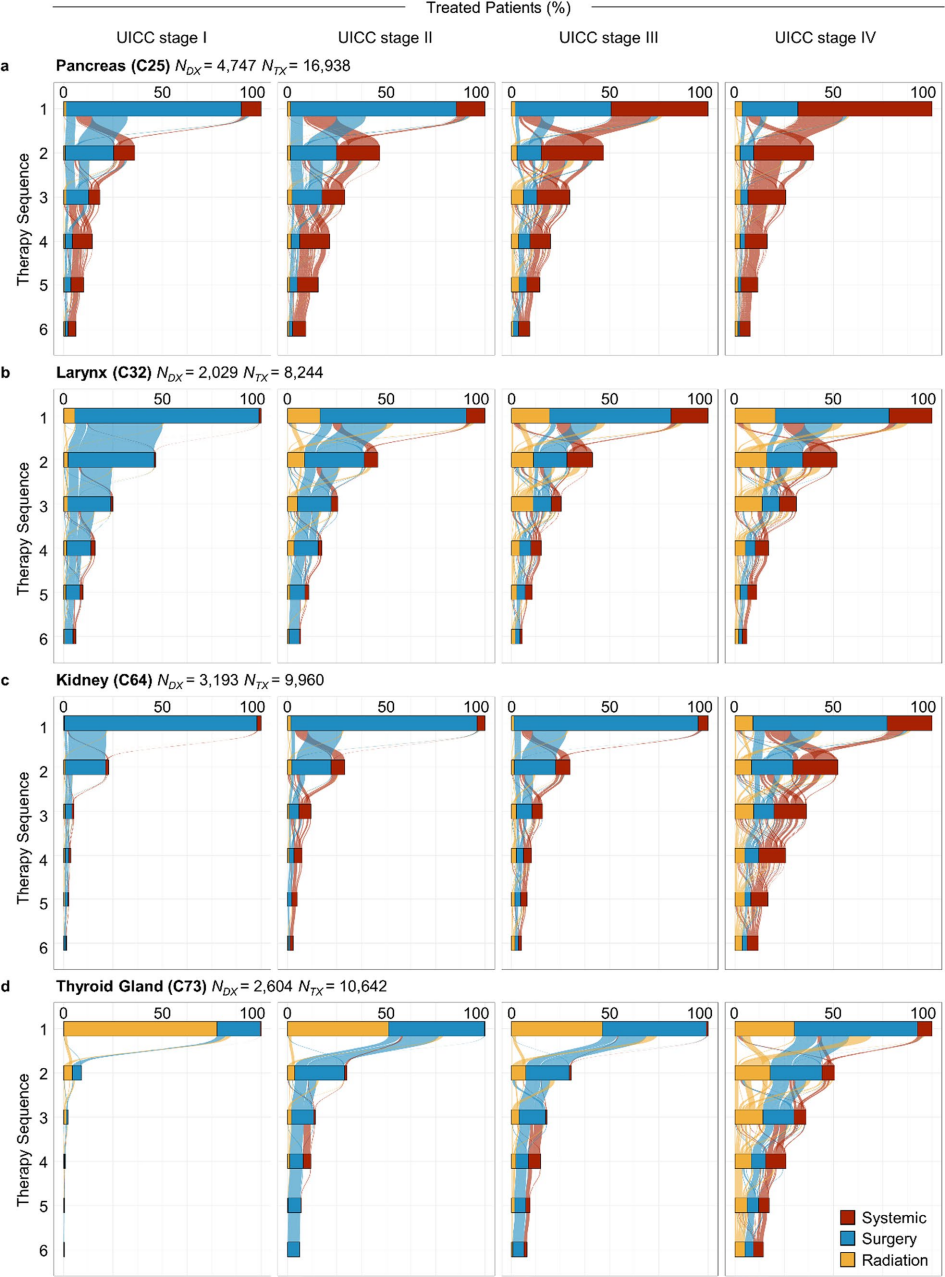
Alluvial diagram of mode-of-therapy sequences stratified by diagnosis and UICC stage for cancer of the **a** pancreas, **b** larynx, **c** kidney and **d** thyroid gland. Illustrated are the percentage of patients who received therapy (x-axis) from the first up to the sixths therapy sequence (bars at y-axis) including the flows of patients (colored alluvial connections) between the different sequences and therapies. *Notes* Number of diagnoses (*N*_*DX*_) according to ICD-10 and therapeutic events (*N*_*TX*_) in the sub-cohort

Figure 5 presents an analysis of therapy sequences faceted by the four disease-specific sub-cohorts and stratified by UICC stage at diagnosis. The visualization illustrates the number of patients who received therapies (x-axis) from the first up to the sixth anti-cancer treatment (y-axis) including the flows of patients between the different modes of therapy. Surgery is the most frequent mode of therapy for early-stage cancer of the pancreas, larynx and kidney (UICC stage I and II). Higher clinical stages at diagnosis (UICC stage III and IV) were more frequently treated with other modalities, which differed among cancers. While systemic therapy dominated in patients with advanced-stage pancreatic cancer, radiation and systemic therapies prevailed in patients with laryngeal or kidney cancer. For cancer of the thyroid gland in early stage (UICC stage I and II), nuclear medicine treatment, such as radioactive iodine therapy, and in later stages, surgery and systemic therapy are most frequently documented. The decreasing size of the frequency bars in Fig. 5 indicate the successive relative reduction of patients receiving additional therapies. This observation holds for all disease-specific sub-cohorts and all strata. The flow of patients between therapies is indicated by the colored alluvial connections between the bars. These alluvial connections illustrate that for early stage (UICC stage I and II) malignancies the mode of the first therapy (e.g., surgery) is re-applied when successive therapy is required. For malignancies detected in later stages (UICC stage III and IV), however, the alluvial streams reflect multimodal anti-cancer-treatment, more often moving from one mode of therapy to another (e.g., from surgery to radiotherapy in stage IV thyroid cancers).

## Discussion and limitations

The present report profiles the pan-cancer multicenter cohort of the DKTK’s CCP which contains 232,911 patients. CNS, non-Hodgkin lymphoma and hepatobiliary cancers ranked higher (with respect to diagnosis frequency and coverage) in the cohort as compared to the national population of cancer patients. We also find high coverage of rare malignancies such as malignant tumors of bones and articular cartilage, cancer of endocrine glands, cancer of the placenta or connective and soft tissue sarcomas. Taken together, these findings indicate a cluster of specialized care in our network of tertiary cancer centers. However, the frequency distribution of the remaining diagnoses in the cohort resembled the distribution in the national population, supporting the assumption that the cohort can in part be considered representative. The cohort features a continuous influx of patients that allows monitoring of their clinical pathways and outcomes. The sharp decline in patients with primary diagnosis in 2021 may be a direct result of the coronavirus pandemic, e.g., because infection control measures delayed elective diagnosis.

Disease-specific sub-cohorts, for which we exemplified diagnosis- and treatment-related analyses, provide detailed insights mirroring known properties of the respective diseases. Our findings concerning the stage-distributions are in line with existing data. For example, early-stage pancreatic cancer is often asymptomatic and thus remains undetected for longer periods of time, which may be considered a reason why most diagnoses find pancreatic cancer in advanced stage [23]. Likewise, documented pancreatic cancer diagnoses predominantly comprise ductal adenocarcinoma, originating from the exocrine pancreas, which is by far more frequent compared to cancer of endocrine origin [24]. Also, for laryngeal cancer, for example, the data are in line with findings from epidemiological cancer registries [25].

While the cohort may serve to monitor clinical outcomes in cancer patients, a recent national research project shows that improved clinical outcomes are positively associated with treatment and treatment options in specialized cancer centers [26]. It must also be considered that university hospital patients are more often part of clinical trials. This circumstance may affect outcomes because clinical trials often require histopathological proof and molecular analysis of the tumor; such deep phenotyping techniques subsequently allow more often for personalized treatment approaches [27, 28].

In summary, the granularity and size of the cohort data is a potential catalyst of translational cancer research. It provides rapid access to comprehensive patient groups of interest and may enhance the understanding of the clinical history of various (even rare) malignancies. Consequently, the cohort may justify decisions in clinical trial design and will contribute to the evaluation of scientific findings under real-world conditions. Moreover, with the application of analytic scripts, data evaluation and visualization can be performed rapidly.

The cohort clearly benefits from its underlying IT-infrastructure, which may serve as a core to future extension of data elements (e.g., laboratory values, genetic information, comorbidities, co-medication, medical history, radiological imaging data) if required for specific research purposes. It enables researchers to access a rich source of harmonized data across the participating sites of the consortium without impairing privacy regulations and the data sovereignty of the hospitals. As a complement to epidemiological data of cancer patients with near complete coverage [23], the cohort of the DKTK’s CCP bridges big data clinical epidemiology and deep-insight real-world cancer research. The cohort dataset is also connected to liquid and tissue biosamples stored in local biobanks allowing to unfold the translational potential of the multi-center pan-cancer cohort.

Of course, the facility-based nature of the cohort data also has its limitations. While certified cancer centers document the corresponding follow-up examinations and ex-domo treatments of their patients, there is not yet an equivalent level of comprehensive documentation of patient journeys treated in non-certified units. Thus, for non-certified cancers, the proportion of covered follow-up and ex-domo treatment events can be expected to be inferior as compared to respective certified diagnoses. The here applied validity assessment in the disease-specific sub-cohorts is limited to face validity, i.e., the plausibility of the data given what we know about disease epidemiology and treatment approaches. In order to strengthen data validity, future assessments should include the predictive validity of the data [4]. Another limitation concerns the specification of inclusion criteria: While the here presented pan-cancer cohort profile was limited to patients diagnosed in 2013 or later and a minimum of two documented disease-related events, these criteria must be re-considered for research focused on specific diseases. For example, a study about long-term survival in prostate cancer patients—a disease for which many German cancer centers have been certified since 2008—would reasonably include patients diagnosed in 2007 onwards and would exclude patients with less than a specified minimum of follow-up examinations. Unlike patient demographics and diagnostic information, it must also be considered that not all data elements can be used directly for analyses. Other data elements must traverse intricate preprocessing in advance of analysis. For example, treatment-related information can be used for the inference of lines of therapy, which might be a useful component to normalize patient data.

In conclusion, we have demonstrated the cohort of the DKTK’s CCP is a patient population representative for German university medicine-based tertiary cancer centers, providing valuable insights into the real-world study of contemporary oncological treatment and outcomes in Germany. Access to biobanks and other data sources build a broad basis for future comprehensive and in-depth analyses.

## Footnotes

1 German CCCs were set up in the mid-2000s; these multidisciplinary cancer care and research facilities built up biobanks and databases to support and facilitate access to high-quality biosamples and comprehensive patient course documentation data. The establishment of comprehensive facility-based documentation of patient courses serves various purposes such as external certification, internal quality assessment and scientific translational and clinical research [7].

2 As the publicly available database provides only statistics for the years 2013–2018, incident cases for the years 2019 and 2020 were estimated as the moving average of the respective past six years.

3 The validity assessment focuses on *face validity*, i.e., the extent to which the presented data are plausible given what we know about the diseases’ epidemiology and treatment approaches [20].

4 Multiple diagnoses of neoplasms in a patient were merged according to rules developed by the International Agency for Research on Cancer, the International Association of Cancer Registries and the European Network of Cancer Registries [22].

5 In this step, patients were excluded if the first date of metastasis detection or the date of the first assessment or treatment was documented prior to the date of the primary diagnosis. Such inconsistencies may not necessarily be the result of incorrect documentation but may enter the data collection if primary diagnosis and/or treatment was conducted in another hospital. Patients for which inconsistencies have been detected were excluded due to insufficient completeness of the documentation of their course of disease.

6 With the enactment of the Cancer Screening and Registry Act, Germany has invested in the development of cancer registration and reimburses for the reporting of registrable events to health care providers. In addition, since that time, many health care institutions, especially large university hospitals, have invested in the certification of their oncology centers, which in turn requires rigorous tumor documentation.

7 Cohort rank is derived from count of diagnoses as indicated in Table 1 (column *Number of patients*). Population rank is derived from the ordered frequency of incident cases registered in the database of the German Center for Cancer Registry Data.
